# Comparison of pharmacy student performance in a self-care therapeutics course conducted as a flipped classroom on-campus and remotely

**DOI:** 10.1186/s12909-023-04581-x

**Published:** 2023-08-22

**Authors:** Bernadette Cornelison, Beth Zerr

**Affiliations:** 1 Pharmacy Practice and Science, University of Arizona R. Ken Coit College of Pharmacy, 1295 N Martin Ave, Tucson, 85721 AZ USA; 2 Pharmacy Practice and Science, University of Arizona R. Ken Coit College of Pharmacy, 605 E Van Buren St, Phoenix, 85004 AZ USA

**Keywords:** Therapeutics, Pharmacy students, Teaching method, Self-care, Online learning, Course performance

## Abstract

**Background:**

The COVID-19 pandemic required the University of Arizona R. Ken Coit College of Pharmacy’s Self-Care Therapeutics course to be taught as a synchronous, live online course. The course has traditionally utilized a flipped-classroom to increase student engagement and improve learning performance. The goal of this study is to compare student performance in a flipped-classroom self-care therapeutics course taught to students attending class on-campus versus online via web-conferencing.

**Methods:**

This study assessed examination performance of 118 students that took the class on-campus in 2019 and 125 students that took the class online in 2020. Course design was similar between the two cohorts, with each completing assigned pre-reading, an associated short multiple-choice quiz, in-class small group discussions and in-class large group faculty-led debrief. Both cohorts took pre-class quizzes and three examinations to assess their knowledge. Exam, quiz, overall class performance, and student experience was compared for the 2019 on-campus attending cohort and the 2020 online attending cohort.

**Results:**

No statistical differences were seen in the overall exam performance, the final course score, and the student experience between cohorts. Statistical differences (p = 0.02) were found between cohorts for the overall quiz performance, with the on-campus attending cohort performing slightly better than the online attending cohort (mean score of 88% compared to 84.4%).

**Conclusion:**

Examination performance was similar for students taking a flipped-classroom course online and on-campus. Further research using data from multiple courses or from the same cohort, randomized, is needed to improve the internal and external validity of these findings.

## Background

The COVID-19 pandemic required many higher education institutions to swiftly change from in-person to online learning. Over 80% of schools of pharmacy reported transitioning at least part of their curriculum online in a 2021 survey of 46 schools [1]. Due to student demand and ongoing health concerns, it is reasonable to predict that many of these schools will continue to offer online components, even after the public health need for remote learning has waned. Schools of pharmacy will still be responsible for delivering quality education, and this change in course modality requires faculty to redesign courses for effective online learning [2]. Online delivery of traditional lecture-based courses confers the risk of decreased student engagement, [3] which can negatively impact learning [4]. Courses that include active learning strategies, including the flipped-classroom modality are designed to encourage student engagement, improve learning performance, [5] and have the potential to mitigate some of the negative aspects of online course delivery [3]. The value of the flipped-classroom delivered in-person has been demonstrated in health professions education [5–7]. There is less available research comparing student performance in flipped-classrooms delivered online in pharmacy education and the literature is even more sparse in therapeutics and self-care pharmacy coursework [3].

A 2022 systematic literature review evaluated the use of a flipped-classroom design in higher education programs that were required to transition to online delivery during the COVID-19 pandemic [3]. This review highlighted the lack of research available assessing online flipped-classroom delivery, specifically across a diverse range of educational programs, and acknowledged the need for more data regarding learning and student performance in online flipped-classrooms. Eighteen studies were analyzed, including one study in pharmacy [3]. The pharmacy study examined student perception and stress but did not evaluate student performance [8]. The three studies that did evaluate student performance found that students performed similarly in flipped-classrooms delivered in-person and online. These studies were conducted with medical students in the United States, postgraduate students in China, and undergraduate chemistry students in the Netherlands [9–11]. Overall, this review found an increase in the use of a flipped-classroom design as institutions attempted to maintain or promote student engagement while also mitigating potential negative effects of remote course delivery [3].

Student demand for remote learning has the potential to affect which institutions students apply to as schools of pharmacy need to remain competitive to attract applicants. Post-pandemic, students have expressed a desire for flexibility with regards to course delivery in higher education, including more options for online learning [12]. Applications to Doctor of Pharmacy (PharmD) programs in the US have decreased steadily over the past 12 years, with 109,150 applicants in 2009 compared to 40,552 in 2021 [13]. During the same timeframe the number of PharmD degrees awarded increased from 11,487 to 2009 to 14,223 in 2021 [14]. Providing opportunities for flexible course delivery is one way to recruit student applicants, but is only feasible if this delivery method still produces a high quality learning experience with comparable educational outcomes. This highlights the need for more research on pharmacy student performance in non-traditional course delivery methods.

The goal of this study is to compare student performance in a self-care therapeutics course, delivered in a flipped-classroom modality, taught to students attending class on-campus versus online.

## Methods

This study utilized a retrospective comparison between on-campus and online flipped-classroom self-care therapeutics courses. The University of Arizona R. Ken Coit College of Pharmacy has utilized a flipped-classroom in its Self-Care Pharmacotherapeutics course since 2018.

Prior to 2020, first year pharmacy students were required to attend a 16-week course on-campus class (August-December 2019) covering 27 self-care therapeutics topics. Prior to class, students were required to complete assigned pre-reading and create study guides using faculty-designed learning objectives as a framework. Students were assigned reading from the Handbook of Nonprescription Drugs [15]. Learning objectives were provided to the students to facilitate their reading and the creation of a study guide. At the beginning of each class, students completed a short multiple-choice quiz on the required reading to assess acquired content knowledge. Students were incentivized to create a study guide by allowing them to use it during the quiz. Quiz scores were provided to the students upon completion of the quiz; however, answers to the quiz questions were provided during class discussions. During class, students self-selected 3–4 peers to work with on patient cases. Questions associated with the Pharmacist’s Patient Care Process (PPCP) were provided to students to answer during small group work [16]. For example, when the students were assessing the patient, the following questions were asked: What is the patient’s primary problem? Does this patient meet exclusion criteria for the primary problem? And is self-care appropriate for this patient? Students were not required to stay in the same group for the semester. This allowed the groups to vary throughout the semester based on student preference. Following small group discussions, faculty led the class through a structured debrief of each presented patient case. Individual preparation, small group peer discussion, and large group student-faculty interaction are all integral components of this course. Three on-campus examinations were administered to the students throughout the semester. Each examination covered nine different self-care topics. Each examination included 33 questions, worth 3 points each, plus one additional extra credit point for a total of 100 points. The examination questions were patient cases that required the students to apply the PPCP and content learned from the pre-work and class. The examination questions were evaluated by three pharmacy educators for accuracy, readability, and difficulty. An item analysis was conducted on each examination to evaluate the validity of the assessment using a Kuder-Richardson 20 (KR-20) score and point biserials [17]. The examination was administered via an online, secure assessment software that ensures that the examination taker only has access to the examination. Other than quiz and examination points, there were no other assessments that were associated with points utilized in the courses.

In the Fall semester of 2020 (August-December 2020), the College of Pharmacy’s Self-Care Therapeutics course was offered as an online course to accommodate for the COVID-19 pandemic. The class was offered as a synchronous live online course to maintain the three integral components of the course: individual preparation, small group peer discussions, and large group faculty interaction. Pre-class individual preparation remained the same as in previous semesters. In-class small group discussions were achieved by randomly placing students in online breakout rooms (3–4 students/group) within the larger virtual class meeting. Large group faculty interaction was achieved via structured question and answer sessions, utilizing audio conferencing, chat functions and online response polling. Students took three examinations throughout the semester. The examinations were also administered via an online, secure assessment software. Examinations were proctored using an online device (such as a phone) connected to a remote proctor via video-conference to monitor and record the student taking the exam on a second online device (such as a computer). The content of covered material did not change between the in-person and online cohorts. Students evaluated the course using a student course survey provided by the University. The only significant difference between the two semesters was the modality of course delivery. Similarities and differences between the design of the course are outlined in Table 1.

**Table 1:** Characteristics of the 2019 and 2020 self-care therapeutics course

	2019 Cohort	2020 Cohort
Length of course	16 weeks	16 weeks
Pre-class work	Assigned readings and creation of study guides	Assigned readings and creation of study guides
In-class Readiness Assurance Test	Multiple choice quiz	Multiple choice quiz
In-class application exercise	Patient cases	Patient cases
Small group discussions (3–4 students/group)	Student, self-selected groups	Random online breakout rooms
Student attendance	In person, on campus	In person, online via Zoom
Exams (3 total)	In person, on campus using an online, secure assessment software	In person, online using an online, secure assessment software
Exam Proctoring	In person proctors	2nd device logged into Zoom

Examination, quiz, and overall class performance was compared for the 2019 on-campus attending cohort and the 2020 online attending cohort. Descriptive statistics were calculated for the on-campus and online attendance cohorts. Summary statistics and boxplots were used to determine whether groups differed in mean. For testing difference in means for the examination and quiz performance, the Mann Whitney Utest was used. For the overall examination and class performance, a two-sample t-test was used to test for the difference between the means. Data provided from the student course surveys were used to compare the student experience. A chi-square test was used to evaluate the 8 questions asked. A significance level of 0.05 was used. All analyses were performed using R Statistical Software (version 4.2.1; R Foundation for Statistical Computing, Vienna, Austria). The need for informed consent was waived by the Institutional Review Board because of the retrospective nature of the study (IRB #20,210,630).

## Results

This study assessed examination performance of 118 students that took the class in 2019 and 125 students that took the class in 2020. Baseline characteristics of the two cohorts are described in detail in Table 2. There were no statistical differences seen between the student cohorts.

**Table 2:** Characteristics of student cohorts

Student Characteristics	On-campus Attendance (2019)	Online Attendance (2020)	P value
Gender, *n (%)*			0.77
Female	82 (69)	89 (71)	
Male	36 (31)	36 (29)	
Admissions data*			
Arizona Resident, *n(%)*			0.41
Yes	112 (93)	114 (90)	
No	8 (7)	12 (10)	
Average Age of Class	24 (20)	23 (18)	n/a
Underrepresented minority in pharmacy, *n(%)*			0.6
Yes	33 (28)	31 (25)	
No	87 (72)	95 (75)	
Students with bachelor’s degree, *n(%)*			0.76
Yes	47 (39)	47 (37)	
No	73 (61)	79 (63)	
Average Cumulative GPA	3.4	3.4	n/a
Average PCAT Composite Score	63.2	62	n/a

*Demographic data collected at admissions

PCAT = Pharmacy College Admission Test

The results for the outcomes of interest are shown in Table 3.

**Table 3:** Class Performance (Exams, Quizzes, and Final Course Score)

	Fall 2019 – on campus (n = 118)	Fall 2020 - remote (n = 125)	P-Value
**Exam 1**			0.02
Min (%)	49	52	
Max (%)	100	100	
Mean (%)	77.2	80.6	
SD	0.11	0.11	
KR20	0.7	0.6	
Point biserial median (range)	0.32 (0.1 to 0.44)	0.26 (0.09 to 0.63)	
**Exam 2**			0.11
Min (%)	61	52	
Max (%)	100	97	
Mean (%)	82	79.4	
SD	0.09	0.11	
KR20	0.5	0.7	
Point biserial median (range)	0.27 (0.03 to 0.45)	0.28 (0.01 to 0.56)	
**Exam 3**			0.95
Min (%)	58	52	
Max (%)	97	100	
Mean(%)	80.9	81	
SD	0.09	0.1	
KR20	0.5	0.6	
Point biserial median (range)	0.24 (0.04 to 0.44)	0.26 (0.02 to 0.44)	
**Overall Exam Performance**			0.77
Min (%)	61	56	
Max (%)	95	98	
Mean(%)	80	80.3	
SD	0.08	0.08	
**Overall Quiz Performance**			0.02
Min (%)	65	61	
Max (%)	100	100	
Mean(%)	88	84.4	
SD	0.06	0.06	
**Final Course Score**			0.89
Min (%)	66	62	
Max (%)	94	98	
Mean(%)	82	81	
SD	0.07	0.07	

KR20 = Kuder-Richardson 20

SD = Standard deviation

**Fig. 1:**
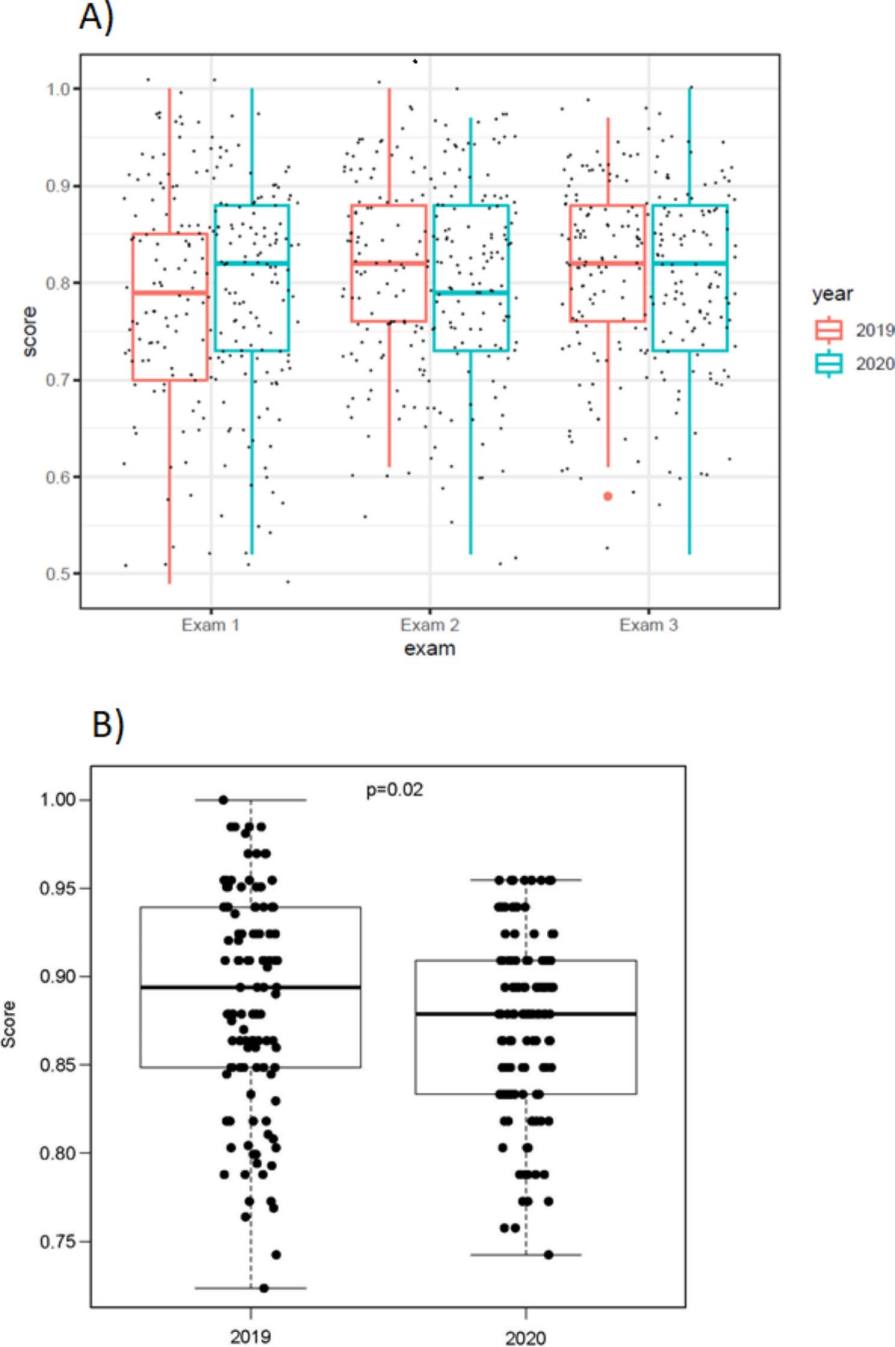
**(A)** Boxplot of student performance on exams. **(B)** Boxplot of overall quiz performance

The summary statistics and boxplot (Fig. 1) show little difference in means between examination performance for 2019 and 2020. No statistical differences were seen in the means between examinations 2 (p = 0.11) and 3 (p = 0.95), overall examination performance (p = 0.77), and the final course score (p = 0.89). Although little difference in means, there were statistical differences between cohorts for examination 1 (p = 0.02) and the overall quiz performance (p = 0.02).

Details of the student experience is outlined in Table 4. No statistical differences were observed between the cohorts for each of the questions asked.

**Table 4:** Evaluation of the student experience based on results received from the student course surveys

Question	Cohort	Strongly Agree (%)	Agree (%)	Uncertain (%)	Disagree (%)	Strongly Disagree (%)	P value
In this course, I was encouraged to participate through class activities, projects, and/or assignments.	2019	64	35	5	3	0	0.60
	2020	58	37	3	1	1	
This course expanded my knowledge and skills in the subject matter.	2019	55	34	6	4	1	1.00
	2020	54	35	7	3	1	
I was encouraged to analyze and/or apply the concepts and skills taught in this course.	2019	49	38	10	2	2	0.49
	2020	51	38	6	5	1	
This course helped me to connect the concepts and skills we learned to the world around me.	2019	49	38	6	4	3	0.97
	2020	48	40	5	5	2	
I feel I learned the subject matter well enough to help another student in this course.	2019	29	43	17	8	3	0.67
	2020	32	34	20	8	5	
The course presentations, materials, procedures, and deadlines were clearly organized.	2019	40	45	4	6	4	0.16
	2020	35	40	14	7	3	
I regularly/frequently had the opportunity to ask questions about concepts and skills in this course.	2019	45	45	6	4	1	0.69
	2020	40	43	11	6	1	
The course material and activities (D2L site, assigned readings, presentations, etc.) helped me learn in this course.	2019	39	46	8	4	2	0.85
	2020	37	43	11	7	2	

## Discussion

In response to the COVID-19 pandemic, faculty aimed to create an online experience that would be comparable to the established, on-campus flipped-classroom course. Student performance, as measured by overall examination scores and the final course score, was similar regardless of attendance method between the two cohorts. Students may have performed similarly due to the design of the course. Other than the course being offered online, the most notable change made to the course was the small group discussions. When the course was offered on campus, students typically chose their classmates to work through patient cases. Online, students were randomized into new small groups every class, and it was rare that the students would work with the same group each class. This may have affected student comfort level with colleagues resulting in less engagement in the small groups. Otherwise, the course design did not change significantly. This study reinforced literature demonstrating similar student performance in flipped courses that were transitioned from in-person to online. Previous studies that examined this include a study in US medical students [9] and a study in Netherlands Information Communications and Technology students [10] that both reported similar performances on final examinations given to an in-person and online cohort. A study conducted with Chinese education students found similar student performance on individual written assessments and group activities [11]. This study supports these results by examining a new population given quizzes and three examinations throughout the semester. Given the change to an online, synchronous course, no differences were observed between the overall examination and the final course score. The small difference that was observed between the two cohorts in examination 1 may have been due to the format that the examination was administered. Students in the online cohort were asked to utilize a non-traditional proctoring method which may have led to additional test anxiety for the first examination [18]. Students should have been prepared similarly for class, however, there was a small difference observed between the quiz scores, with the on-campus cohort performing slightly better. This could have been due to the online cohort experiencing different extrinsic conditions, related to COVID-19, that did not allow them to prepare as well as the cohort that was taught in-person.

Similar to other studies examining online flipped classrooms in higher education, [8–10] student experience did not significantly differ between the online and on-campus cohorts. The demonstration of consistent student experience further supports the similarity of course design between the two cohorts.

### Limitations

One of the main limitations to this study is the difficulty in comparing two separate class cohorts that were experiencing very different extrinsic conditions. The on-campus cohort took the class during the Fall of 2019, prior to the pandemic, and the online cohort took the class during the Fall of 2020, after the COVID-19 pandemic necessitated the need for the transition to online schooling. This undoubtedly affected student performance due to pandemic-related stress including, but not limited to, learning online, risk of getting sick, losing family members, and having children at home. Although the baseline characteristics of both cohorts were similar, comparing two different cohorts is a limitation of this study as we were unable to account for baseline covariates. Additionally, the multiple-choice examinations were the sole major assessments delivered in this course. Best practices for online learning typically involve summative and formative assessments. This can be done by using a pre-test with incorporated feedback which is matched with a post-test. Other practices used include peer feedback and self-reflection [2]. Positive student performance results could mean the student is a “good guesser” or that they do well at taking multiple choice examinations. As a result, the examinations alone may not reflect students’ understanding of the content [18]. Finally, differences in how examinations were delivered and proctored in the on-campus vs. online cohorts may have affected examination scores.

Anecdotally, some students did report inconsistent group participation in the online cohort. This is similar to results found in US chemistry undergrad students who reported a lack of comfort speaking up in their online flipped course during small group work [3]. If the participation in small groups was decreased, this may indicate that the value of small group work in this course is not as integral to student success as previously thought. The small group work is intended to have the students apply the pre-course work to a patient. The large group discussion is designed to review the patient cases with the intent of reiterating important points and clarifying misunderstandings discussed in the small groups. The online small groups required the students to turn on their camera and microphone to interact with their classmates in breakout rooms. Engagement during small group discussions could be gauged in the on-campus cohort, however, the online virtual platform did not allow for this in a non-disruptive manner. The students in the online cohort may not have had as much engagement in the small groups as the students that were in-person. Further iterations of this course may need to consider restructuring the small group work.

## Conclusion

This research study demonstrates that overall examination and class performance was similar for students taking a self-care therapeutics course as a flipped-classroom online and in-person. Further research using data from multiple courses or from the same cohort, randomized, is needed to improve the internal and external validity of these findings.

## Data Availability

The datasets generated and/or analysed during the current study are not publicly available due to it being student related data but are available from the corresponding author on reasonable request.
